# Live-Birth Prediction of Natural-Cycle *In Vitro* Fertilization Using 57,558 Linked Cycle Records: A Machine Learning Perspective

**DOI:** 10.3389/fendo.2022.838087

**Published:** 2022-04-22

**Authors:** Yanran Zhang, Lei Shen, Xinghui Yin, Wenfeng Chen

**Affiliations:** ^1^Medical School of Nanjing University, Nanjing, China; ^2^College of Computer and Information, Hohai University, Nanjing, China; ^3^ Nanjing Marine Radar Institute, Nanjing, China

**Keywords:** NC-IVF, HFEA, machine learning, ensemble learning, live birth

## Abstract

**Background:**

Natural-cycle *in vitro* fertilization (NC-IVF) is an *in vitro* fertilization (IVF) cycle without gonadotropins or any other stimulation of follicular growth. Previous studies on live-birth prediction of NC-IVF were very few; the sample size was very limited. This study aims to construct a machine learning model to predict live-birth occurrence of NC-IVF using 57,558 linked cycle records and help clinicians develop treatment strategies.

**Design and Methods:**

The dataset contained 57,558 anonymized register patient records undergoing NC-IVF cycles from 2005 to 2016 filtered from 7bsp;60,732 records in the Human Fertilisation and Embryology Authority (HFEA) data. We selected matching records and features through data filtering and feature selection methods. Two groups of twelve machine learning models were trained and tested. Eight metrics, e.g., F1 score, Matthews correlation coefficient (MCC), the area under the receiver operating characteristic curve (AUC), etc., were computed to evaluate the performance of each model.

**Results:**

Two groups of twelve models were trained and tested. The artificial neural network (ANN) model performed the best in the machine learning group (F1 score, 70.87%; MCC, 50.37%; and AUC score, 0.7939). The LogitBoost model obtained the best scores in the ensemble learning group (F1 score, 70.57%; MCC, 50.75%; and AUC score, 0.7907). After the comparison between the two models, the LogitBoost model was recognized as an optimal one.

**Conclusion:**

In this study, NC-IVF-related datasets were extracted from the HFEA data, and a machine learning-based prediction model was successfully constructed through this largest NC-IVF dataset currently. This model is universal and stable, which can help clinicians predict the live-birth success rate of NC-IVF in advance before developing IVF treatment strategies and then choose the best benefit treatment strategy according to the patients’ wishes. As “use less stimulation and back to natural condition” becomes more and more popular, this model is more meaningful in the decision-making assistance system for IVF.

## Introduction

Infertility is defined as failure to achieve a clinical pregnancy after 12 months of regular and unprotected sexual intercourse (1–4). Assisted reproductive technologies (ARTs), especially *in vitro* fertilization (IVF) and embryo transfer (ET) (IVF-ET), are advanced technologies to help infertile couples get pregnant (5, 6). IVF can be divided into natural-cycle IVF (NC-IVF) and stimulated IVF (SIVF) according to whether ovarian stimulation is used or not in the process of IVF. NC-IVF is an IVF cycle without gonadotropins or any other stimulation of follicular growth, and it leads to only one follicle development in most cases. However, SIVF is an IVF cycle that uses gonadotropin stimulation to generate many follicles to improve chances of conception and pregnancy success (7). The world’s first successful IVF pregnancy occurred after NC-IVF in 1978 (8). Since then, SIVF has been widely used in IVF treatment (9). At the same time, NC-IVF is only used as an alternative to SIVF and only for patients with poor ovarian responder, advanced age, religious reasons, etc. (10, 11). However, NC-IVF has its unique and irreplaceable advantages: і) NC-IVF treatment can be performed every month without daily injections, luteal phase support, and adjuncts to improve endometrial function. ii) The endometrium in NC-IVF will not be negatively affected by supraphysiological estradiol concentration (12, 13). iii) Cryopreservation of zygotes or embryos and discarding of surplus embryos is not required in NC-IVF treatment. iv) NC-IVF treatment has no ovarian hyperstimulation syndromes (OHSS) and rare multiple pregnancies (14). v) The implantation rate per oocyte collected during NC-IVF is higher than that in SIVF, and the embryo quality is also better in NC-IVF (15–17). vi) NC-IVF treatment has a lower cost per cycle and better perinatal outcomes (18; 19–21). vii) The average psychological treatment distress is lower in NC-IVF treatment (22–24). Briefly, NC-IVF is a low-risk, low-cost, and patient-friendly treatment procedure (25).

However, unfortunately, for the purpose and consideration of obtaining as many follicles as possible, doctors generally give priority to recommending SIVF treatment in the current clinical treatment. But even experienced doctors can hardly guarantee which treatment will benefit patients more. If there is a prediction method that can predict the live-birth occurrence using NC-IVF treatment after entering the basic information of patients, can it assist clinicians in developing treatment strategies?

Up till now, reliable and accurate prediction of IVF outcomes has always been an outstanding issue. Meanwhile, applying computational prediction models should be an optimized solution. Computational prediction models estimate the future treatment outcome and offer recommendations by analyzing a variety of related features. With the rapid improvement of computer technology, artificial intelligence (AI) has been explosively developed. Machine learning is an application of AI. It extracts the features of data, trains its capability to analyze features, and develops prediction models based on accumulated experience (intermediate results). Machine learning-based prediction models are increasingly used in clinical decision-making, mostly in complex multi-variable systems (26–28).

**Figure 1:**
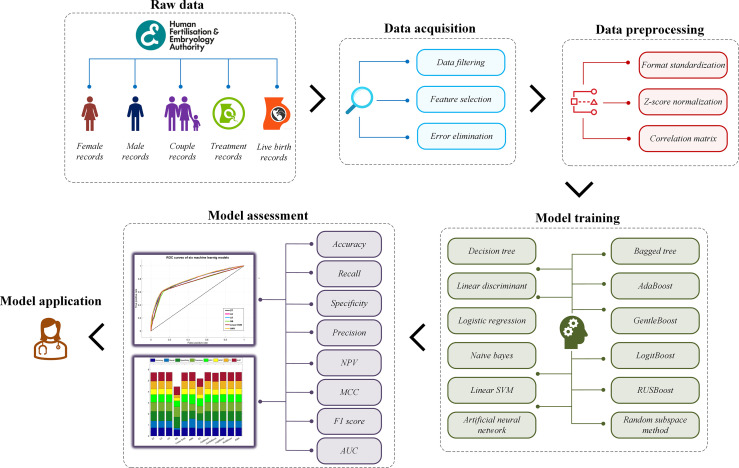
The overall model building framework.

This study aims to construct a prediction model for live-birth occurrence of NC-IVF, using a comprehensive, varied dataset of 57,558 anonymized register cycle records undergoing NC-IVF cycles from 2005 to 2016 filtered from 760,732 records in the Human Fertilisation and Embryology Authority (HFEA) dataset. Two groups of a total of twelve machine learning models were trained and tested using the dataset. The model construction mainly includes four steps. Step 1: acquire and prepare a dataset, which is the combination of selected and filtered patient records. Step 2: pre-process dataset using specific algorithms to standardize the format, normalize the data, and select features. Step 3: train prediction models with machine learning algorithms in two groups. Step 4: evaluate the performance of each model and find the best one. The overall model building framework is shown in **Figure 1**.

## Materials and Methods

### Data Acquisition

The dataset was obtained from the HFEA, which collected data and statistics about the fertility treatment cycles performed each year in the United Kingdom. HFEA holds the longest-running register of fertility treatments data in the world to improve patient care and help researchers to conduct world-class research while ensuring very strong protection of patient, donor, and offspring confidentiality. The raw dataset in this study contained 760,732 cycle records with 95 fields on treatment cycles started between 2005 and 2016. This study mainly focused on the prediction of live-birth occurrence, so the “Live birth occurrence” field was regarded as the prediction label, while the other 94 fields were regarded as features. Each record represented patients’ situations in one cycle. All data were anonymized and freely available on their website (https://www.hfea.gov.uk/), so no ethics approval was required for this study.

First of all, couples undergoing IVF [including intracytoplasmic sperm injection (ICSI)] were considered. “Egg donation,” “Sperm donation,” “Embryo donation,” and “Surrogate” were excluded. Secondly, in IVF, exogenous gonadotropins are used to stimulate the development of more than one egg at a time, which is called “Stimulation used” in the raw dataset (7, 29, 30). So patients who had no “Stimulation used” (i.e., undergoing NC-IVF) were considered in this study. Then, fresh cycles and the following frozen–thawed cycles from NC-IVF were all included. Furthermore, cycles with completed ET were included. Finally, after some records containing outliers such as “999” were eliminated, 57,558 cycle records were retained for further analysis, i.e., 57,558 NC-IVF cycles.

The raw dataset contains 94 features. Obviously, not all features contributed significantly to predicting live-birth occurrence. As our prediction model was designed as a pretreatment model to predict the live-birth occurrence of the couple before the IVF treatment started, features related to “Egg retrieval,” “Egg stored,” “Fertilization,” “Embryo transfer,” and “Embryo stored” were excluded. On the contrary, features related to “Patient age,” “Previous pregnancy status of the couple,” “Previous pregnancy related treatments,” “Type of infertility,” “Cause of infertility,” “Treatment type,” “Fresh cycle,” and “Frozen cycle” were included. After these steps, 34 features were selected. A detailed description of selected features is summarized in **Table 1**.

**Table 1:** Description of 35 fields in the dataset.

Field name	Field type	Description
Patient Age at Treatment	Categorical	Patient age at treatment, banded as follows: 18–34, 35–37, 38–39, 40–42, 43–44, 45–50.
Total Number of Previous Treatments, Both IVF and DI at Clinic	Numeric	The number of treatment cycles of IVF and DI the patient has previously had at the clinic associated with this treatment.
Total Number of Previous IVF Cycles	Numeric	The number of treatment cycles of IVF the patient has previously had.
Total Number of Previous DI Cycles	Numeric	The number of treatment cycles of DI the patient has previously had.
Total Number of IVF Pregnancies	Numeric	Times the patient has been pregnant through IVF.
Total Number of DI Pregnancies	Numeric	Times the patient has been pregnant through DI.
Total Number of Live Births—Conceived through IVF	Numeric	The number of live births the patient has had through IVF.
Total Number of Live Births—Conceived through DI	Numeric	The number of live births the patient has had through DI.
Type of Infertility—Female Primary	Categorical	1 if the patient has never been pregnant, 0 otherwise.
Type of Infertility—Female Secondary	Categorical	1 if the patient has ever been pregnant, 0 otherwise.
Type of Infertility—Male Primary	Categorical	1 if the partner has never impregnated any woman, 0 otherwise.
Type of Infertility—Male Secondary	Categorical	1 if the partner has ever impregnated some woman, 0 otherwise.
Type of Infertility—Couple Primary	Categorical	1 if the patient has never been pregnant while the partner has never impregnated any woman, 0 otherwise.
Type of Infertility—Couple Secondary	Categorical	1 if the patient has ever been pregnant while the partner has ever impregnated some woman, 0 otherwise.
Cause of Infertility—Tubal Disease	Categorical	1 if the primary cause of infertility is due to tubal disease, 0 otherwise.
Cause of Infertility—Ovulatory Disorder	Categorical	1 if the primary cause of infertility is due to ovulatory disorder, 0 otherwise.
Cause of Infertility—Male Factor	Categorical	1 if the primary cause of infertility is due to the partner, 0 otherwise.
Cause of Infertility—Patient Unexplained	Categorical	1 if the primary cause of infertility is unknown, 0 otherwise.
Cause of Infertility—Endometriosis	Categorical	1 if the primary cause of infertility is due to endometriosis, 0 otherwise.
Cause of Infertility—Cervical Factors	Categorical	1 if the primary cause of infertility is due to cervical factors, 0 otherwise.
Cause of Infertility—Partner Sperm Concentration	Categorical	1 if the primary cause of infertility is due to partner sperm concentration, 0 otherwise.
Cause of Infertility—Partner Sperm Morphology	Categorical	1 if the primary cause of infertility is due to partner sperm morphology, 0 otherwise.
Causes of Infertility—Partner Sperm Motility	Categorical	1 if the primary cause of infertility is due to partner sperm motility, 0 otherwise.
Cause of Infertility—Partner Sperm Immunological Factors	Categorical	1 if the primary cause of infertility is due to partner sperm immunological factors, 0 otherwise.
Main Reason for Producing Embryos Storing Eggs	Categorical	The main reason for storing eggs in this cycle and producing embryos in subsequent cycles, includes treatment now, for storing eggs.
Specific Treatment Type	Categorical	The specific treatment type used in this cycle includes IVF and ICSI.
PGD	Categorical	1 if this cycle involved the use of preimplantation genetic diagnosis, 0 otherwise.
PGD Treatment	Categorical	1 if this cycle would be contained in the “PGD” CaFC category on the HFEA website, 0 otherwise.
PGS	Categorical	1 if this cycle involved the use of preimplantation genetic screening, 0 otherwise.
PGS Treatment	Categorical	1 if this cycle would be contained in the “PGS” CaFC category on the HFEA website, 0 otherwise.
Elective Single Embryo Transfer	Categorical	1 if this cycle involved the deliberate use of only one embryo, 0 otherwise.
Fresh Cycle	Categorical	1 if this cycle used fresh embryos, 0 otherwise.
Frozen Cycle	Categorical	1 if this cycle used frozen embryos, 0 otherwise.
Embryos Transferred	Numeric	The number of embryos transferred into the patient in this cycle.
Live-Birth Occurrence	Categorical	1 if there were one or more live births as a result of this cycle, 0 otherwise.

IVF, in vitro fertilization; DI, donor insemination; ICSI, intracytoplasmic sperm injection; HFEA, Human Fertilisation and Embryology Authority.

### Data Preprocessing

The data format was standardized into two types: numeric and categorical, e.g., “Patient age at treatment,” “Type of infertility,” “Cause of infertility,” “Specific treatment type,” and “Live birth occurrence” were categorical (i.e., two categories: 0 and 1; multiple categories: 0, 1, 2, 3…), and “Total number of previous IVF cycles,” “Total number of live births,” and “Total number of IVF pregnancies” were numeric.

The normalization method *via* Z-score was then applied as a pre-processing step to all features (31). Feature vectors were normalized *via* Z-score normalization, as follows:


$$X_{norm}=\frac{X-\mu }{\sigma }$$

where *x* is the feature vector, *x_norm_* is the normalized vector, *μ* is the mean value of the feature vector, and *σ* is the standard deviation of that. Hence, all feature vectors have a mean of 0 and a standard deviation of 1. After the Z-score normalization step, the classification performance of models would not be affected by the value range of data.

**Figure 2:**
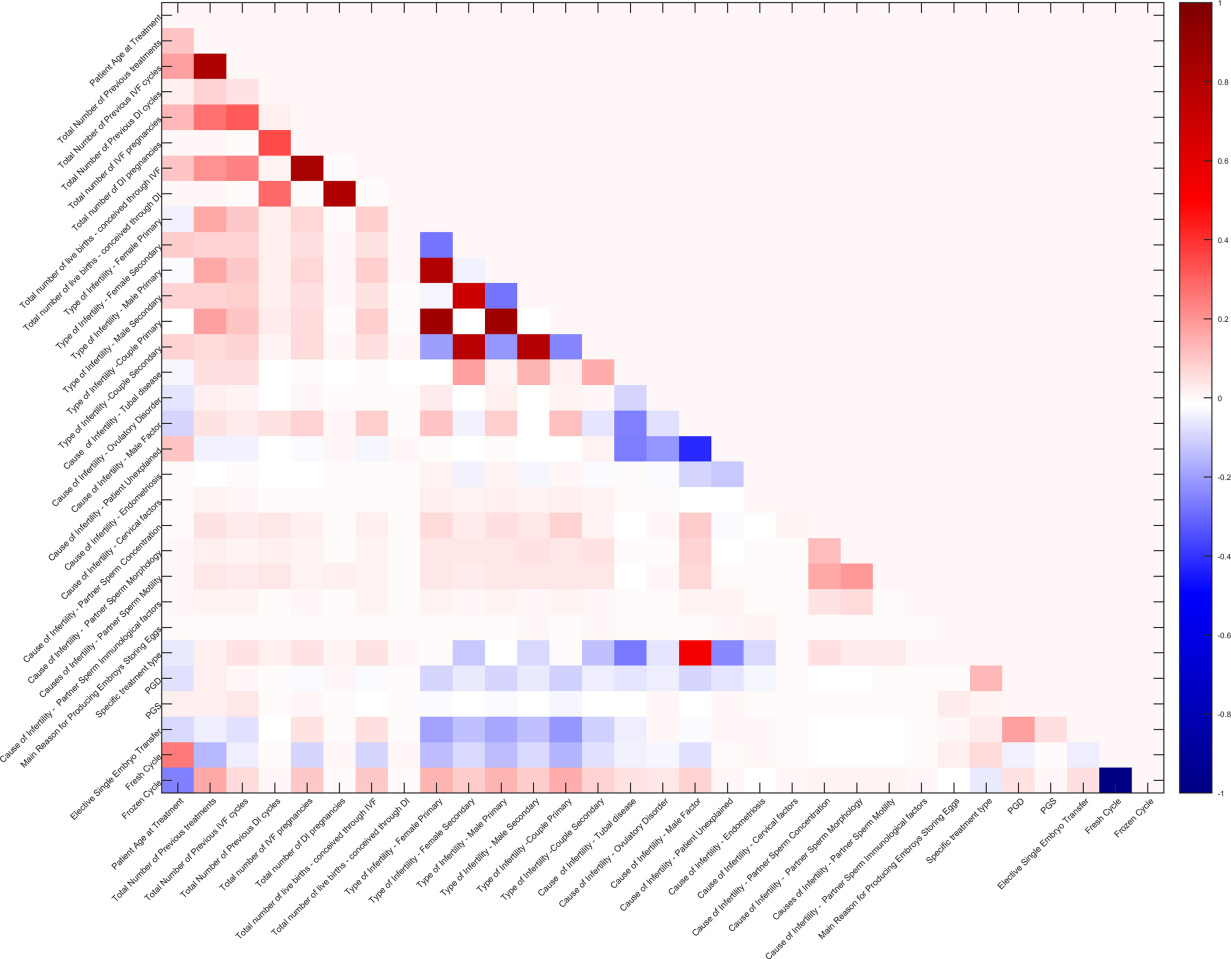
Correlation matrix of 31 features. The redder grids indicate higher positive correlation values of feature pairs, the bluer ones indicate higher negative correlation values, and the white ones indicate no correlation.

In addition, Pearson’s correlation coefficients between 34 features were also calculated. Pairs with a correlation coefficient higher than a threshold (close to 1) were reduced to only one as the input of the model. The correlation matrix of 31 features (after reduction) is shown in **Figure 2**.

### Model Training

After preprocessing, the dataset contained 57,558 samples, including a 57,558 × 31 feature matrix and a 57,558 × 31 label vector. Among the 57,558 samples, the number of positive samples (i.e., live birth was true) was 12,340, the number of negative samples was 45,218, and the negative samples were nearly four times the positive ones. Obviously, there was a certain degree of imbalance in the dataset. The normal processing methods for dataset imbalance include the following: і) keeping all positive samples, but down-sampling negative samples, and ii) keeping all negative samples and over-sampling positive samples. The former method will bring a lot of information loss, while the latter one will cause a sharp increase of training dataset and the over-fitting problem (32). Since the imbalance ratio was not large, and the complexity of model training should not be increased, in this study, the dataset was divided into 4 sub-datasets at a ratio of 1:1, and each sub-dataset contained all positive samples and 1/4 negative samples (the negative samples in the fourth sub-dataset partially overlapped with others, and those in other three sub-datasets were independent with each other). Decision tree (DT) and linear discriminant (LD) algorithms were used to pre-train the four sub-datasets, and then four groups of evaluation metrics including precision, recall, and F1 score were obtained. The sub-dataset with the best score was selected as the dataset for further model training. After this step, the dataset to be used had 24,680 samples, containing exactly the same number of positive and negative samples.

In this study, twelve models were selected, trained, tested, and analyzed. They were divided into two groups: і) machine learning models, i.e., independent classifiers, included DT, LD, logistic regression (LR), naive Bayes (NB), linear support vector machine (Linear SVM), and artificial neural network (ANN); ii) ensemble learning models, i.e., combined classifiers, including bagged tree (BT), AdaBoost, GentleBoost, LogitBoost, RUSBoost, and random subspace method (RSM).

DT algorithm builds classification or regression models in the form of a tree structure. It breaks down a dataset into smaller and smaller subsets, while at the same time an associated DT is incrementally developed. The final result is a tree with decision nodes and leaf nodes. The topmost decision node in a tree that corresponds to the best predictor is called the root node (33). LD algorithm, also known as Fisher’s LD (FLD), is a classic algorithm for pattern recognition. The basic implementation method is to project high-dimensional samples into the best discriminant vector space to achieve the effect of extracting classification information and compressing the dimension of the feature space (34). LR algorithm produces a logistic curve, which is limited to values between 0 and 1. The curve is constructed using the natural logarithm of the “odds” of the target variable, rather than the probability (35). NB algorithm is based on Bayes’ theorem with the independence assumptions between predictors. An NB model is easy to build, with no complicated iterative parameter estimation, which makes it particularly useful for very large datasets (36). SVM algorithm is a kind of generalized linear classifier that helps to identify the maximum-margin hyperplane for the positive and negative classes as a decision boundary. In addition, the SVM algorithm can perform non-linear classification through the kernel method: using a kernel function to map the original training samples to a high-dimensional space. The ANN attempts to recreate the computational mirror of the biological neural network. There are different types of neural networks but are generally classified into feed-forward and feed-back networks. A feed-forward network is a non-recurrent network that contains inputs, outputs, and hidden layers; the signals can only travel in one direction. Input data are passed onto a layer of processing elements where it performs calculations. Each processing element makes its computation based upon a weighted sum of its inputs. The new calculated values then become the new input values that feed the next layer. This process continues until it has gone through all the layers and determines the output (37, 38). In this study a two-layer feed-forward network, with sigmoid hidden and softmax output neurons, was used to classify pattern vectors (live-birth label vector), given 20 neurons in its hidden layer. This network was trained with scaled conjugate gradient backpropagation.

Ensemble learning is a kind of technology that combines a variety of compatible machine learning algorithms/models to perform a single task in order to obtain better prediction performance. Ensemble learning is generally classified into three types: bagging, boosting, and stacking. In this study, two bagging methods (i.e., BT and RSM) and four boosting methods (i.e., AdaBoost, GentleBoost, LogitBoost, and RUSBoost) were built and tested. The bagging method is based on multiple sub-datasets divided through the bootstrap algorithm. Then, multiple models are trained, and the best one is selected using the voting method. In this study, a DT was used as the classifier for the method, so this model was called tree-based bagging or BT (39). RSM, also known as feature bagging, trains each classifier by using some random features instead of all features to reduce the correlation between each classifier (40). In this study, LD was used as the classifier for the RSM model. The boosting algorithm combines a series of weak classifiers into a strong classifier to improve performance. The Adaboost algorithm uses class probability estimates to construct real-valued contributions of the weak classifiers, LogitBoost is an adaptive Newton algorithm by stagewise optimization of the Bernoulli likelihood, GentleBoost is an adaptive Newton algorithm *via* stagewise optimization of the exponential loss, RUSBoost is an algorithm combining random undersampling, and RUSBoost is especially for unbalanced datasets (41–43).

The complete training and validation analysis was implemented *via* MATLAB software (R2020a, Natick, MA, USA). The auxiliary debugging tools were also developed for recording the performance during model training in this environment.

### Assessment Method

A standard validation method was essential to evaluate the performance of each model. In this study, a 10-fold cross-validation method was used to assess the robustness of each model. The dataset was randomly divided into 10 equal-sized subsets, and the cross-validation process was repeated 10 times. Each time, one of the 10 subsets was used as the validation set for testing the model, and the remaining nine subsets were put together to form a training data set. Finally, 10 results of experiments were averaged to produce a single estimation for each model.

The performance of the models was evaluated in terms of common standard machine learning evaluation metrics (44). These metrics were computed based on the values of true negatives (TN), true positives (TP), false positives (FP), and false negatives (FN) as detailed below.


$$Accuracy=\frac{TP+TN}{TP+FP+TN+FN}$$

$$Recall=\frac{TP}{TP+FN}$$

$$Specificity=\frac{TN}{TN+FP}$$

$$Precision\left(PPV\right)=\frac{TP}{TP+FP}$$

$$NPV=\frac{TN}{TN+FN}$$

$$MCC=\frac{TP*TN-FP*FN}{\sqrt{\left(TP+FP\right)*\left(TN+FN\right)*\left(TP+FN\right)*(TN+FP)}}$$

$$F1score=2*\frac{Precision*Recall}{Precision+Recall}$$

In addition, confusion matrix plots can help to understand how the currently selected model performed in each class and identify the areas where the model performed poorly (45). The area under the receiver operating characteristic curve (AUC-ROC) (46), which also represents the overall performance of model and prediction, has the value ranging from 0 to 1, where 1 represents the best performance and 0 is the worst performance, and AUC = 0.5 means random classification.

## Results

### Study Population

A total number of 57,558 NC-IVF cycles (samples) were selected in the HFEA dataset from 2005 to 2016. A total of 12,340 cycles resulted in positive live births, while 45,218 cycles resulted in negative live births. Among the 12,340 positive-live-birth cycles, 5,570 received IVF, accounting for 45.14%, and 6,770 received ICSI, accounting for 54.86%. By comparison, among the 45,218 negative-live-birth cycles, 21,539 received IVF, accounting for 47.63%, and 23,679 received ICSI, accounting for 52.37%. The age distribution of all patients undergoing NC-IVF is as follows: 18- to 34-year-old patients accounted for the largest proportion, reaching 42.79%; it is followed by 35- to 37-year-old patients, accounting for 24.84%; and the least was 45- to 50-year-old patients, accounting for 1.24%. In the “Type of infertility” category, “Couple primary” accounted for the largest proportion, reaching 34.11%, while “Couple secondary” had the least proportion, reaching 11.08%. In the category of “Cause of infertility,” the top five were “Male factor” (38.09%), “Patient unexplained” (26.28%), “Tubal disease” (18.85%), “Ovulatory disorder” (14.47%), and “Endometriosis” (5.80%). More detailed statistics are listed in **Table 2**.

**Table 2:** Baseline characteristics of NC-IVF cycles.

Characteristic	NC-IVF cycles 2005–2016 (n = 57,558)			
	Positive live birth (n = 12,340)		Negative live birth (n = 45,218)	
	n	%	n	%
*Patient age at treatment (year)*				
18–34	6,222	50.42	18,409	4.09
35–37	3,211	26.02	11,089	24.52
38–39	1,642	13.31	6,824	15.09
40–42	1,060	8.59	6,478	14.33
43–44	163	1.32	1,747	3.86
45–50	42	0.34	671	1.48
*Type of infertility*				
Female primary	3,186	25.82	13,642	30.17
Female secondary	1,667	13.51	7,597	16.80
Male primary	3,171	25.70	13,515	29.89
Male secondary	1,655	13.41	7,608	16.83
Couple primary	3,678	29.81	15,867	35.09
Couple secondary	1,151	9.33	5,227	11.56
*Cause of infertility*				
Tubal disease	2,029	16.44	8,818	19.50
Ovulatory disorder	1,844	14.94	6,484	14.34
Male factor	4,916	39.84	17,007	37.61
Patient unexplained	3,191	25.86	11,933	26.39
Endometriosis	725	5.88	2,614	5.78
Cervical factors	6	0.05	27	0.06
Partner sperm concentration	47	0.38	235	0.52
Partner sperm morphology	40	0.32	144	0.32
Partner sperm motility	23	0.19	116	0.37
Partner sperm Immunological factors	2	0.02	5	0.01
*Specific treatment type*				
IVF	5,570	45.14	21,539	47.63
ICSI	6,770	54.86	23,679	52.37

IVF, in vitro fertilization; ICSI, intracytoplasmic sperm injection.

### Model Assessment and Comparison

**Figure 3:**
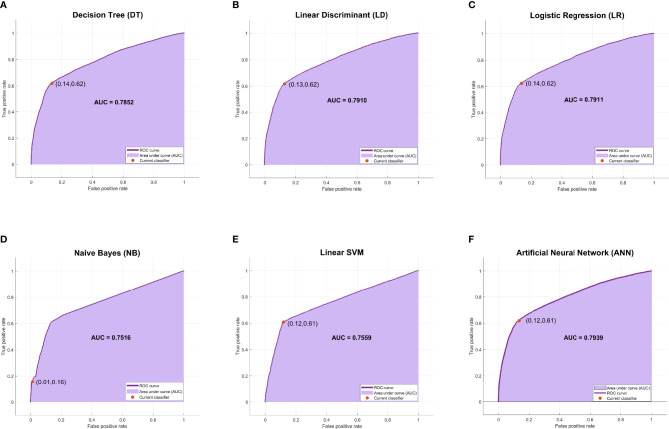
The ROC curves and AUC scores of six machine learning models. **(A)** The ROC curve and AUC score of the DT model: the deep purple curve refers to the ROC curve, the area under the curve is covered by light purple color, the orange dot represents the threshold that corresponds to the optimal operating point, and the AUC score is clearly marked. **(B–F)** The ROC curve and AUC score of LD, LR, NB, Linear SVM, and ANN, respectively. ROC, receiver operating characteristic; AUC, area under the receiver operating characteristic curve; DT, decision tree; LD, linear discriminant; LR, logistic regression; NB, naive Bayes; ANN, artificial neural network.

The evaluation metrics of all models were compared in terms of accuracy, recall, specificity, precision, negative predictive value (NPV), Matthews correlation coefficient (MCC), and F1 score as listed in **Table 3**. Among the six machine learning models, ANN, LR, and LD models achieved the best F1 scores (70.87%, 70.82%, and 70.68%, respectively). As shown in **Figure 3**, ANN, LR, and LD models also obtained the best AUC scores (0.7939, 0.7911, and 0.7910, respectively). Although the scores of the three models were very close, obviously, the ANN model performed the best in terms of metrics.

**Table 3:** Evaluation metrics of all models.

Model	Accuracy	Recall	Specificity	Precision	NPV	MCC	F1
**Machine learning models**							
DT	74.19	61.90	86.47	82.06	69.42	49.90	70.57
LD	74.44	61.62	87.26	82.87	69.45	50.57	70.68
LR	74.34	62.27	86.42	82.10	69.59	50.17	70.82
NB	57.14	15.57	98.72	92.40	53.90	25.72	26.65
Linear SVM	74.38	60.72	88.04	83.54	69.15	50.69	70.33
ANN	74.42	62.24	86.61	82.30	69.64	50.37	70.87
**Ensemble learning models**							
BT	67.78	72.88	62.69	66.14	69.80	35.75	69.34
AdaBoost	74.37	61.33	87.41	82.97	69.33	50.49	70.53
GentleBoost	73.85	62.87	84.82	80.55	69.55	48.88	70.62
LogitBoost	74.47	61.22	87.72	83.29	69.34	50.75	70.57
RUSBoost	74.33	61.22	87.44	82.98	69.28	50.43	70.46
RSM	74.41	61.28	87.54	83.11	69.33	50.60	70.54

The values in the table represent percentages.

NPV, negative predictive value; MCC, Matthews correlation coefficient; DT, decision tree; LD, linear discriminant; LR, logistic regression; NB, naive Bayes; SVM, support vector machine; ANN, artificial neural network; BT, bagged tree; RSM, random subspace method.

**Figure 4:**
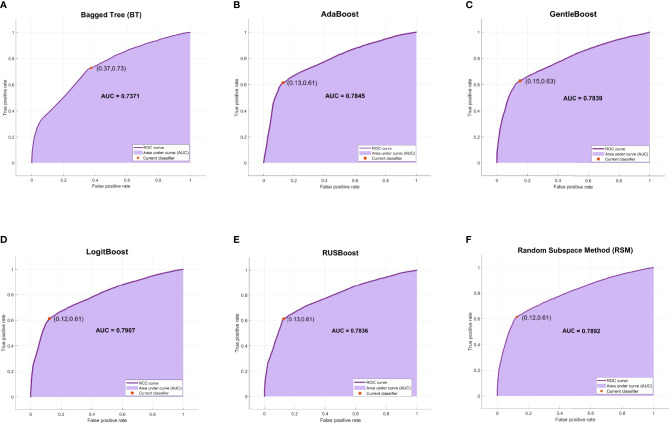
The ROC curves and AUC scores of six ensemble learning models. **(A)** The ROC curve and AUC score of BT model: the deep purple curve refers to the ROC curve, the area under the curve is covered by light purple color, the orange dot represents the threshold that corresponds to the optimal operating point, and the AUC score is clearly marked. **(B–F)** The ROC curve and AUC score of AdaBoost, GentleBoost, LogitBoost, RUSBoost, and RSM, respectively. ROC, receiver operating characteristic; AUC, area under the receiver operating characteristic curve; BT, bagged tree; RSM, random subspace method.

Among the six ensemble learning models, the performance differences were slight except for the BT model. GentleBoost, LogitBoost, and RSM models achieved the best F1 scores (70.62%, 70.57%, and 70.54%, respectively) as listed in **Table 3** and the best AUC scores (0.7839, 0.7907, and 0.7892, respectively) as shown in **Figure 4**. Moreover, LogitBoost obtained another best score, i.e., MCC (50.75%), which is defined as a comprehensive indicator like the F1 score. In summary, the LogitBoost model was considered the best performer after comparison with other ensemble learning models.

**Figure 5:**
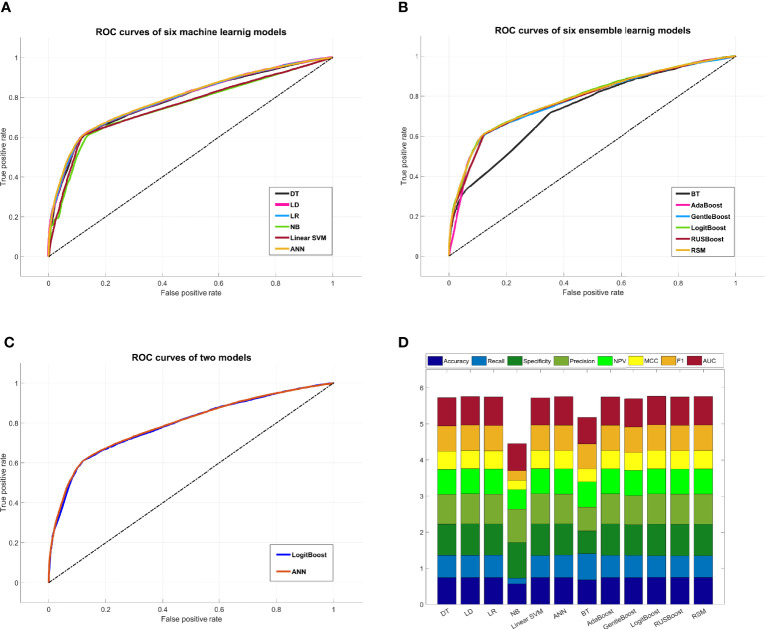
Comprehensive comparison of all models. **(A)** Comprehensive ROC curves of six machine learning models: the larger the area under the curve, the better the performance. **(B)** Comprehensive ROC curves of six ensemble learning models. **(C)** Comprehensive comparison of two ROC curves: ANN model, i.e., the best machine learning model, and LogitBoost model, i.e., the best ensemble learning model. **(D)** In this histogram, the metrics of twelve models, including accuracy, recall, specificity, precision, NPV, MCC, F1, and AUC, are stacked into columns; hence, the higher the column, the better the performance. ROC, receiver operating characteristic; ANN, artificial neural network; NPV, negative predictive value; MCC, Matthews correlation coefficient.

A comprehensive comparison of all models is shown in **Figure 5**. The ROC curve of the ANN model covered the largest area among the six machine learning models in **Figure 5A**, while the ROC curve of the LogitBoost model covered the largest area among the six ensemble learning models in **Figure 5B**. The comparison of ROC curves between the ANN model and the LogitBoost model in **Figure 5C** implied that the performance difference might be very small, and the stacking effect of all metrics could also illustrate this point as shown in **Figure 5D**.

ANN and LogitBoost are two completely different algorithm models. In this study, we found that the two models achieved almost indistinguishable performance. In other words, the two models had almost the same prediction abilities under this specific dataset. Finally, we also included the training time in the performance evaluation. Under the specific computer platform involved in this study, the training time of ANN was about 89.804 s, while that of LogitBoost was about 11.193 s. Obviously, the training efficiency of LogitBoost is higher than ANN, so the LogitBoost model would be considered the optimal model and software would be designed for actual prediction.

## Discussion

Machine learning has become a new discipline, which integrates the application of psychology, biology, neurophysiology, mathematics, automation science, and computer science to form the theoretical basis of machine learning. Currently, machine learning has rapidly demonstrated its ability to predict human fertility (47). So far, the main studies using machine learning models to predict better IVF outcomes are as follows: і) a deep convolutional neural network (CNN) model was trained to assess an embryo’s implantation potential (48). ii) AI technology based on determinant-weighting analysis could offer an individualized embryo selection strategy for any given patient and predict clinical pregnancy rate and twin risk (49). iii) A machine learning algorithm could use clinical parameters and markers of capacitation to predict successful fertilization in normospermic men undergoing IVF (50). iv) A random forest (RF) model was built to predict the implantation potential of a transferred embryo (51). Moreover, the relevant studies using the HFEA dataset for analysis and prediction are as follows: і) a logistic model was fitted to predict the live-birth rate following IVF based on the number of eggs and the age of the female using HFEA data (52). ii) Two clinical prediction models were developed to estimate the individualized cumulative chance of a first live birth over a maximum of six complete cycles of IVF using HFEA data (53). iii) Three clinical models were used to assess live birth and perinatal outcomes with the HFEA database (54).

Whether using AI prediction models or clinical prediction models and whether based on small samples from single-center or big data from expert organizations like HFEA, current studies focused on the associations between IVF outcome and embryo morphology, embryo quality, embryo freezing, etc. The connection between IVF outcome and a different ovarian stimulation was rarely reported. In fact, a different ovarian stimulation directly determines the number of eggs obtained and the egg quality, which are directly related to the embryo quality, and embryo quality is the key factor affecting IVF outcome. Before an actual IVF treatment, what kind of ovarian stimulation is the first choice that the patients need to face. Due to a lack of expertise in patients, the use of ovarian stimulation is mainly based on the recommendation and judgment of clinicians. But even experienced clinicians can hardly guarantee which kind of ovarian stimulation results in a better outcome. IVF technology has only been developed for decades. The long-term potential impact of “stimulation used” on offspring is currently unknown. Today, more and more people are calling for “back to nature,” to simulate the conception process in the natural state as much as possible. It seems to be of great value to predict the outcome of such a natural process.

We creatively incorporated the ovarian stimulation scheme into the basic conditions of big data filtering and research. After selecting the NC-IVF records from the HFEA dataset, which is one of the largest IVF datasets in the world, we built a live-birth prediction model and provided greater precision than previous individual studies. To our knowledge, the dataset in this study is the largest one focusing on NC-IVF by now. The prediction model is universal and stable, which can help clinicians predict the live-birth success rate of NC-IVF in advance before developing IVF treatment strategies and then choose the best benefit treatment strategy according to the patients’ wishes. If the success rate is high, the patients will be recommended to enter an NC-IVF cycle. Otherwise, a SIVF cycle can be considered to generate many follicles to improve chances for conception and pregnancy (7).

The limitations of this study include the live-birth occurrence prediction for cumulative NC-IVF cycles not being studied. As the HFEA data were anonymized cycle-based records rather than patient-based, we were unable to identify patients who had undergone more than one cycle in the dataset. Furthermore, some features, e.g., smoking, body mass index (BMI), the number of good-quality embryos, the total dose of gonadotropins, and the methods of freezing or thawing embryos, have been reported and confirmed to be related to the IVF outcome. As the HFEA data did not include these records, our study was not comprehensive enough. Besides, the HFEA data only represent the anonymized patients from the United Kingdom. If possible, in the future, we will include anonymized data from more institutions, regions, and even more races. This study only focused on NC-IVF cycles. In the next step, the study of SIVF will also be considered to provide a more comprehensive prediction method.

In conclusion, previous studies on live-birth prediction of NC-IVF were very few, the sample size was very limited, and most of the studies were based on women under unfavorable conditions, which might lead to low reliability of results. In this study, NC-IVF-related datasets were extracted from the HFEA data, and a machine learning-based prediction model was successfully constructed through this largest NC-IVF dataset currently. A total of twelve machine learning models were trained, and the best one was selected to predict the live birth. This model is universal and stable, which can help clinicians predict the live-birth success rate of NC-IVF in advance before developing IVF treatment strategies and then choose the best benefit treatment strategy according to the patients’ wishes. We hope that similar models should be promoted so that the IVF treatment can “use less stimulation and back to natural condition,” for the purpose of reducing the burdens and risks of patients and reducing the potential risks of offspring due to stimulation as much as possible.

An application software based on the prediction model will be developed. Once the patient’s data (features) are entered into the software, a prediction result (positive or negative, and probability) will be displayed. Combined with the experience of clinicians, it can obviously assist in decision-making. Therefore, this is the basic idea of an intelligent decision support system. Moreover, with the continuous expansion of the dataset, for example, more cycle records are obtained from the HFEA, the model will be updated to achieve higher accuracy.

## Data Availability Statement

The datasets presented in this study can be found in online repositories. The names of the repository/repositories and accession number(s) can be found below: https://www.hfea.gov.uk/about-us/our-data/#ar.

## Ethics Statement

Ethical review and approval were not required for the study on human participants in accordance with the local legislation and institutional requirements. Written informed consent for participation was not required for this study in accordance with the national legislation and the institutional requirements.

## Author Contributions

YZ and LS initiated and conceived the study. YZ was responsible for data acquisition and data preprocessing. LS was responsible for model construction. YZ and LS wrote the manuscript. XY and WC advised on the development of the study and revised and commented on the draft. All authors read and approved the final version of the manuscript.

## Conflict of Interest

The authors declare that the research was conducted in the absence of any commercial or financial relationships that could be construed as a potential conflict of interest.

## Publisher’s Note

All claims expressed in this article are solely those of the authors and do not necessarily represent those of their affiliated organizations, or those of the publisher, the editors and the reviewers. Any product that may be evaluated in this article, or claim that may be made by its manufacturer, is not guaranteed or endorsed by the publisher.
